# Letrozole Suppresses the Fusion of Osteoclast Precursors through Inhibition of p38-Mediated DC-STAMP Pathway

**DOI:** 10.3390/ijms21218396

**Published:** 2020-11-09

**Authors:** Hyung Joon Kim, Hwa-Sik Seong, YunJeong Choi, Soon Chul Heo, Yong-Deok Kim

**Affiliations:** 1 Department of Oral Physiology, Periodontal Diseases Signaling Network Research Center, and Dental and Life Science Institute, School of Dentistry, Pusan National University, Yangsan 50611, Korea; hjoonkim@pusan.ac.kr (H.J.K.); celinechoi@pusan.ac.kr (Y.C.); snchlheo@gmail.com (S.C.H.); 2 Department of Oral and Maxillofacial Surgery, Dental Research Institute, Periodontal Diseases Signaling Network Research Center, and Dental and Life Science Institute, School of Dentistry, Pusan National University, Yangsan 50611, Korea; mega1024@naver.com

**Keywords:** letrozole, dendritic cell-specific transmembrane protein (DC-STAMP), p38, osteoclast fusion, bone resorption

## Abstract

Letrozole is a reversible nonsteroidal aromatase inhibitor that is widely used in postmenopausal breast cancer patients. It is well established that letrozole decreases bone density owing to estrogen depletion; however, few studies have reported its direct effect on bone cells in vitro. Therefore, we investigated the effect of letrozole on bone metabolism, focusing on osteoclastogenesis. Letrozole did not affect the viability, proliferation, or migration of bone marrow-derived macrophages (BMMs); however, it reduced the multinucleation of immature osteoclasts and subsequent bone resorption in vitro. Overall, letrozole inhibited the expression of dendritic cell-specific transmembrane protein (DC-STAMP), tartrate-resistant acid phosphatase, calcitonin receptor, and cathepsin K. Among them, the reduced expression of DC-STAMP was the most prominent. However, this downregulation of DC-STAMP expression following letrozole treatment was not related to the inhibition of major osteoclastogenesis pathways, such as the nuclear factor-κB (NF-κB), c-Fos, and nuclear factor of activated T cell c1 (NFATc1) pathways, but was attributed to the inhibition of p38, which is known to reside upstream of DC-STAMP expression. Notably, the anti-osteoclastogenic effect of letrozole was abolished following treatment with the p38 activator anisomycin. Contrary to our expectations, these results strongly suggest a previously unknown anti-osteoclastogenic activity of letrozole, mediated by the downregulation of the p38/DC-STAMP pathway.

## 1. Introduction

Bone is a dynamic tissue that maintains homeostasis through the harmonious regulation of bone resorption by osteoclasts and bone formation by osteoblasts [1,2]. An imbalance in bone homeostasis can result in several pathological bone diseases. Osteoporosis can result from excessive bone resorption by osteoclasts, whereas osteosclerosis could occur owing to the increased bone formation by osteoblasts [3].

Osteoclasts are multinucleated giant cells derived from hematopoietic monocyte/macrophage lineage precursors through osteoclastogenesis. An osteoclastogenic stimulus, such as parathyroid hormone (PTH), upregulates the expression of the receptor activator of nuclear factor kappa-B ligand (RANKL) and the macrophage colony-stimulating factor (M-CSF) from osteoblasts and stromal cells, whereas osteoprotegerin (OPG), a well-known decoy receptor for RANKL [3], simultaneously blunts osteoclast differentiation. After the ligation of RANKL and RANK, the receptor for RANKL, sequential osteoclastogenic signaling pathways are triggered, including nuclear factor kappa-B (NF-κB), c-fos, and mitogen-activated protein kinases (MAPKs), such as c-Jun N-terminal kinase (JNK), extracellular signal-related kinase (ERK), and p38 MAP kinase (p38MAPK) [4,5]. These signaling pathways ultimately converge on the expression of the nuclear factor of activated T cell c1 (NFATc1), a master regulator of osteoclastogenesis [6]. NFATc1 not only controls the expression of genes encoding tartrate-resistant acid phosphatase (TRAP) and cathepsin K (CTK) [7] but is also involved in the multinucleation of pre-osteoclasts by cell fusion molecules, such as dendritic cell-specific transmembrane protein (DC-STAMP) and osteoclast transmembrane protein (OC-STAMP) [8,9]. 

Letrozole is a reversible nonsteroidal P450 aromatase inhibitor that prevents the conversion of androgen to estrogen by binding to the heme of its cytochrome P450 unit [10]. It is widely used as a USFDA-approved endocrine therapeutic agent in postmenopausal breast cancer patients to prevent estrogen-induced recurrence and metastasis. In the case of bone metabolism, the most common side effect of letrozole administration in vivo is osteoporosis owing to estrogen depletion. It is well-known that estrogen increases OPG and decreases M-CSF and RANKL, thus resulting in the downregulation of osteoclastogenesis [11,12,13]. However, few studies have focused on the direct effect of letrozole on osteoclastogenesis and bone metabolism. Hence, we investigated the direct effect of letrozole on osteoclast differentiation in vitro.

## 2. Results

### 2.1. Letrozole Demonstrated No Cytotoxicity on Mouse Bone Marrow-Derived Macrophages (BMMs) and Did Not Alter BMM Proliferation

**Figure 1:**
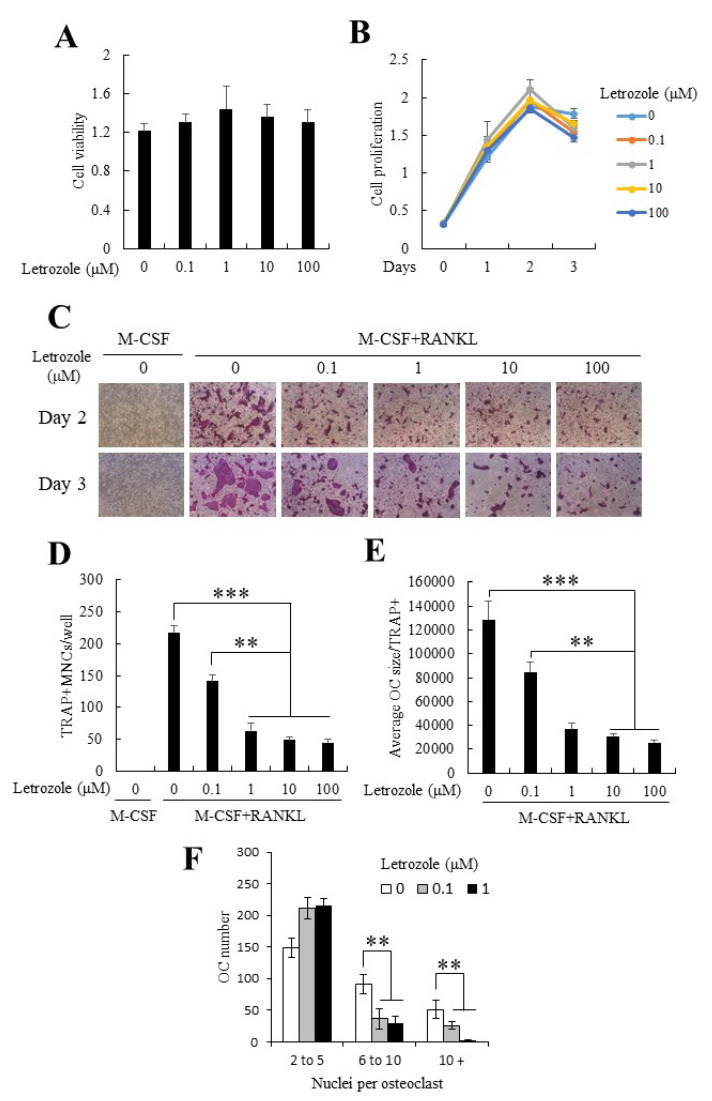
Letrozole suppressed osteoclastogenesis of mouse bone marrow-derived macrophages (BMMs). (**A**) BMMs were cultured in a medium with indicated letrozole concentrations (0–100 µM). After 24 h, cell viability was measured using the 3-(4,5-dimethylthiazol)-2,5-diphenyltetrazolium bromide (MTT) assay, as described in Materials and Methods. (**B**) BMMs were cultured in the osteoclastogenic medium (30 ng/mL M-CSF + 100 ng/mL RANKL) in the presence of letrozole (0–100 μM) for 3 days. Cell proliferation was measured using the MTT assay. (**C**) BMMs were differentiated into osteoclasts in the osteoclastogenic medium with indicated letrozole concentrations (0–100 µM) for 3 days. After culture, tartrate-resistant acid phosphatase (TRAP) staining was performed to visualize osteoclasts. (**D**) TRAP-positive multinucleated cells (TRAP + MNCs) were quantified, and (**E**) the average size of TRAP-positive osteoclasts was measured. (**F**) The numbers of osteoclasts were shown according to the nuclei per one multinucleated osteoclast. The TRAP-positive multinucleated cells with ≥3 nuclei were considered osteoclasts. All quantitative data are presented as mean ± standard deviation (SD), ** *p <* 0.01 *** *p* < 0.001.

In the present study, we first examined the effects of letrozole on cell viability and proliferation using purified primary mouse bone marrow-derived macrophages (BMMs). As shown in Figure 1A,B, letrozole demonstrated no cytotoxicity (Figure 1A) and did not alter cell proliferation at the examined concentrations (0–100 µM; Figure 1B).

### 2.2. Letrozole Inhibited RANKL-Induced Osteoclastogenesis of BMMs

To investigate the effect of letrozole on osteoclastogenesis, BMMs were cultured in M-CSF medium alone as an undifferentiated control group or in an osteoclastogenic medium (30 ng/mL M-CSF + 100 ng/mL RANKL) with indicated concentrations of letrozole for 2 or 3 days. Both groups were stained for TRAP. RANKL administration strongly induced osteoclast differentiation, and the presence of letrozole markedly decreased the formation of TRAP-positive multinucleated cells (TRAP+MNCs; Figure 1D) in a dose-dependent manner. Notably, the anti-osteoclastogenic effect of letrozole was prominent on the reduced osteoclast size; for instance, in the high-dose letrozole-treated group, TRAP-positive osteoclasts were still present, but their size was smaller than that of osteoclasts of the RANKL-only treated group (Figure 1E). Letrozole decreased the number and size of multinucleated osteoclasts in a dose-dependent manner, with almost maximum efficacy, even at a concentration of 1 μM. Furthermore, a significant reduction in the number of nuclei per one multinucleated osteoclast was observed in the presence of letrozole (Figure 1F). These results suggested that letrozole inhibited osteoclast differentiation and reduced the fusion of pre-osteoclasts.

### 2.3. Letrozole Decreased Osteoclastic Bone Resorption But Did Not Affect Cell Migration

**Figure 2:**
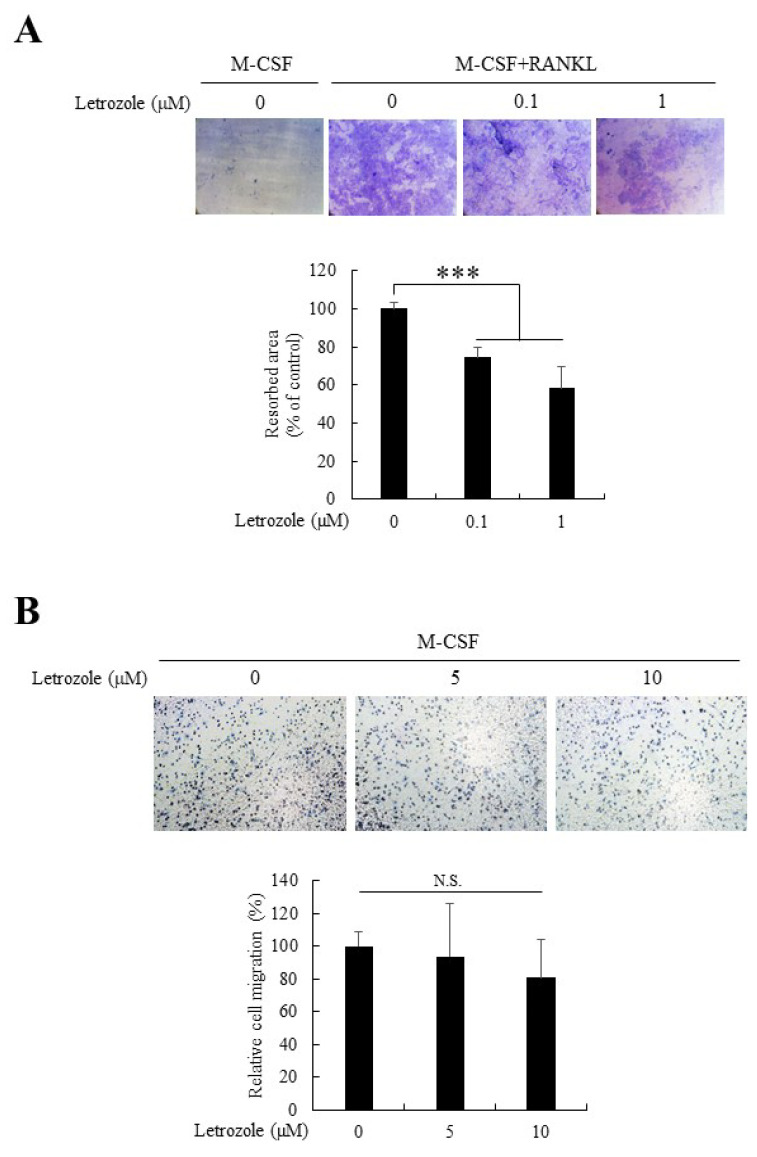
Letrozole decreased the osteoclastic bone resorption activity with minimal effect on the migration of BMMs. (**A**) BMMs were seeded on dentin discs and allowed to differentiate into osteoclast with the osteoclastogenic medium, in the presence of indicated letrozole concentrations (0–1 µM) for 8 days. The resorption pits were visualized by toluidine staining and subsequently quantified using imaging software, as described in Materials and Methods. (**B**) BMMs were plated on the upper compartment of the Boyden chamber (8-µm pore size) and incubated for 16 h in the presence of macrophage colony-stimulating factor (M-CSF); 30 ng/mL). The migrated BMMs on the bottom side of the membrane were stained with hematoxylin and quantified. All quantitative data are presented as mean ± standard deviation (SD), *** *p* < 0.001. N.S.: not significant.

To functionally delineate the suppression of osteoclast differentiation by letrozole further, we performed a resorption pit formation assay by culturing osteoclasts on dentin discs with indicated letrozole concentrations (0–1 μM) for 8 days. As expected, letrozole significantly decreased the bone resorption activity demonstrated by osteoclasts. As shown in Figure 2, 0.1 μM of letrozole was sufficient to reduce bone resorption. Furthermore, we examined the migration activity of BMMs in the presence of letrozole to determine the underlying cause of letrozole-mediated downregulation of pre-osteoclast fusion. However, letrozole did not affect the migration of BMMs (Figure 2B). Therefore, we concluded that the decreased pre-osteoclast fusion upon letrozole treatment could not be attributed to the anti-migratory action of letrozole.

### 2.4. Letrozole Reduced the mRNA Expression of TRAP, CTR, CTK, and DC-STAMP

**Figure 3:**
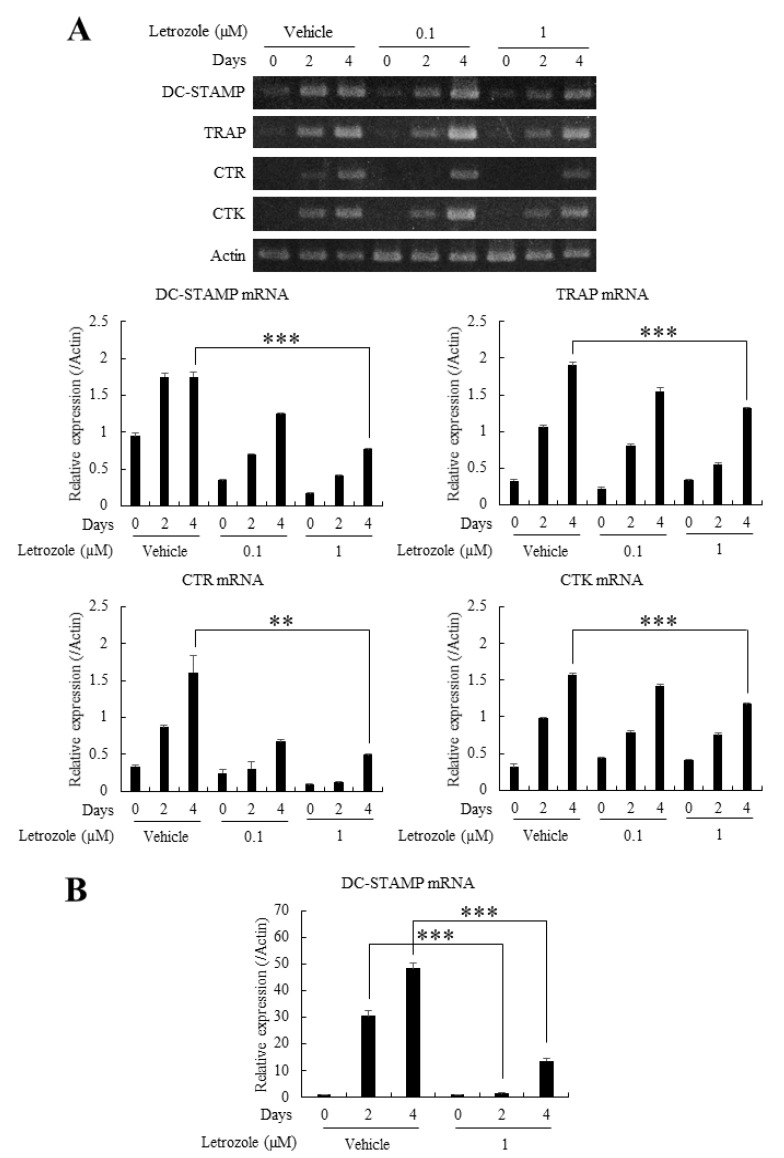
Letrozole reduced the expression of osteoclast marker genes and that of the osteoclast fusion regulator dendritic cell-specific transmembrane protein (DC-STAMP). (**A**) (upper panel) BMMs were incubated in a medium containing M-CSF and receptor activator of nuclear factor kappa-B ligand (RANKL) with indicated letrozole concentrations (0–1 µg) for 4 days. The mRNA expression levels of DC-STAMP, TRAP, CTR, and cathepsin K (CTK) were determined by conventional RT-PCR analyses. (lower panel) The relative band intensities of test markers from reverse transcription-polymerase chain reaction (RT-PCR) performed in triplicates were normalized to that of the control band (actin) and are presented as statistical graphs. (**B**) The mRNA expression level of DC-STAMP was reevaluated by quantitative real-time PCR. All quantitative data are presented as mean ± standard deviation (SD), ** *p* < 0.01 *** *p* < 0.001.

To quantitatively evaluate the anti-osteoclastogenic properties of letrozole, we analyzed mRNA expression levels of several osteoclast marker genes. First, BMMs were cultured in indicated combinations of RANKL (100 ng/mL) or letrozole (0–1 μM) for 4 days and the mRNA expression levels of DC-STAMP, TRAP, CTR, and CTK were determined by conventional reverse transcription-polymerase chain reaction (RT-PCR). A majority of the examined mRNA expression levels showed a declining tendency depending on the concentrations of letrozole, which were highly prominent on day 4 (Figure 3A). In particular, the expression of DC-STAMP, a well-appreciated fusion promoting molecule, also known to enhance osteoclastogenesis [8,9], was noticeably reduced following letrozole treatment. As letrozole decreased both the size of osteoclasts and osteoclast differentiation, the mRNA expression level of DC-STAMP was reevaluated by quantitative real-time PCR, presenting a more conspicuous decrease when compared with conventional RT-PCR (Figure 3B). Thus, we presumed that the reduced pre-osteoclast fusion, as well as osteoclast differentiation, in the presence of letrozole originated from letrozole-induced downregulation of DC-STAMP expression.

### 2.5. Letrozole Did Not Affect the Expression of Osteoclastogenic Transcription Factors, NFATc1, c-Fos, or the Activation of NF-κB

**Figure 4:**
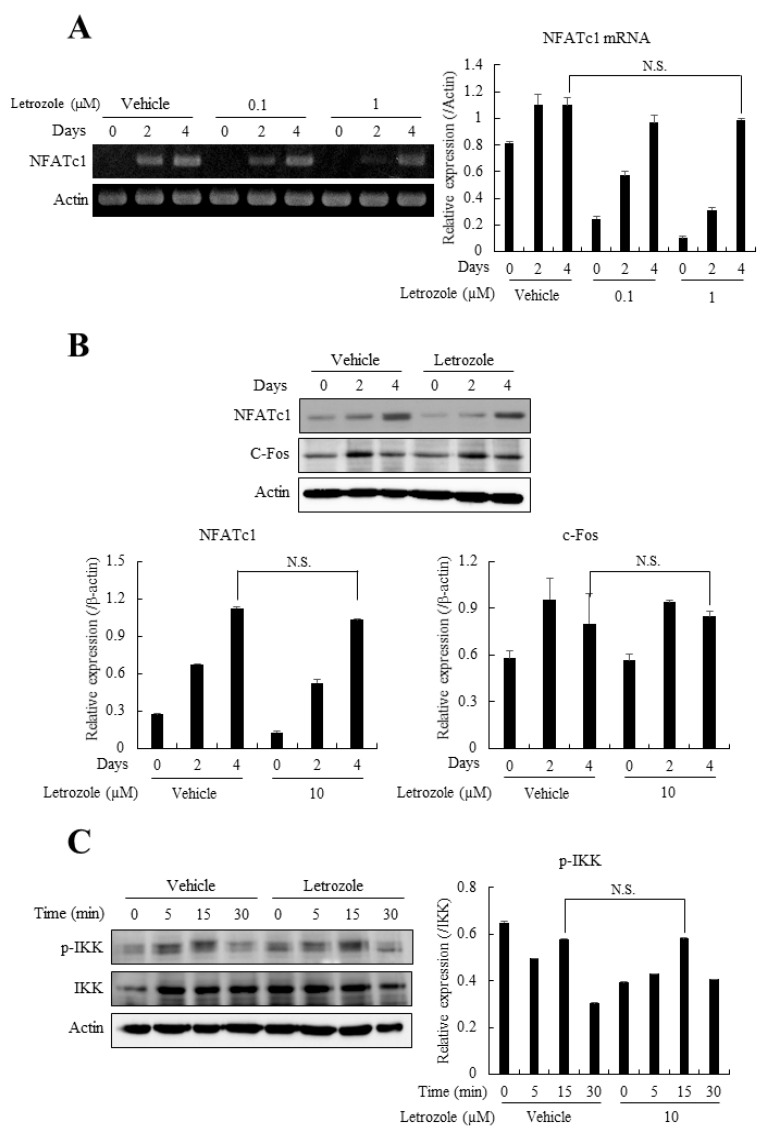
Letrozole did not affect the expression of transcription factors (nuclear factor of activated T cell c1 (NFATc1) and c-Fos) and the activation of the nuclear factor-κB (NF-κB) pathway. (**A**) BMMs were incubated in osteoclastogenic medium (M-CSF + RANKL) with indicated letrozole concentrations (0–1 µM) for 4 days. (left panel) The mRNA level of NFATc1 was evaluated by RT-PCR. (right panel) The relative band intensity of NFATc1 was normalized with the actin band and quantified. (**B**) BMMs were cultured in the osteoclastogenic medium for 2 or 4 days, and protein expressions of c-Fos and NFATc1 were determined by western blotting. (**C**) BMMs were serum-starved for 4 h and pretreated with vehicle (DMSO) or letrozole (10 μM) for another 1 h, followed by stimulation with RANKL (100 ng/mL) for indicated times. Cell lysates were prepared and the phosphorylation of inhibitory kB kinase (IKK) was examined by western blotting. N.S.: not significant.

As letrozole showed the most prominent decrease in DC-STAMP mRNA expression, we next focused on determining the causative pathway for the observed reduction. First, the NF-κB/c-Fos/NFATc1 pathway, the major axis of osteoclastogenesis, was evaluated. The mRNA expression level of NFATc1, examined by RT-PCR, demonstrated non-significant differences even on day 4 (Figure 4A), and the protein expression levels of NFATc1 and c-Fos, as well as the phosphorylation of cytoplasmic inhibitory κB kinase (IKK), showed minimal difference in the presence or absence of letrozole (Figure 4B,C). These results are contrary to our expectations that letrozole may suppress major osteoclastogenic pathways, such as NFATc1 and c-Fos, because these pathways are known to reside upstream of DC-STAMP expression [14,15]. Consequently, we investigated another upstream pathway, focusing on DC-STAMP.

### 2.6. Letrozole Diminished p38 Activation in RANKL-Stimulated BMMs

**Figure 5:**
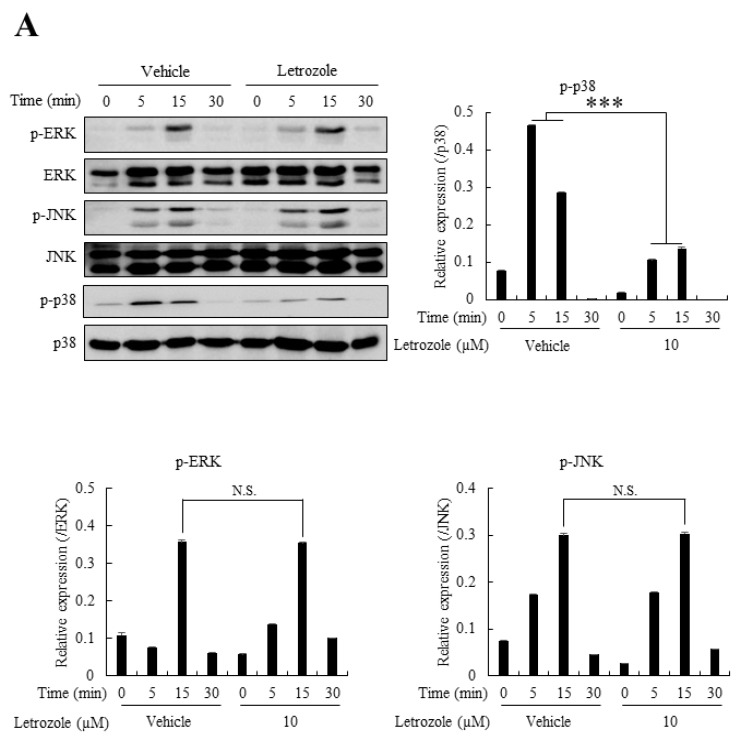
Letrozole demonstrated minimal effects on the phosphorylation of ERK and JNK but reduced the phosphorylation of p38. (**A**) The 4 h serum-starved BMMs were pretreated with an equal volume of vehicle (DMSO) or letrozole (10 μM) for 1 h. RANKL stimulation (100 ng/mL) was performed for indicated times, and cells were harvested for evaluating p-p38, phosphorylation extracellular signal-related kinase (p-ERK), and phosphorylation c-Jun N-terminal kinase (p-JNK) with western blotting. The relative band intensity of phosphorylated proteins was normalized to that of the un-phosphorylated protein and represented as a graph. All quantitative data are presented as mean ± standard deviation (SD), *** *p* < 0.001.

Although the importance of c-Fos and NFATc1 in the direct upregulation of DC-STAMP is widely accepted, recent reports have suggested the existence of a p38 MAPK- mediated DC-STAMP expression pathway, independent of the c-Fos/NFATc1 pathway [8,16,17]. For example, caffeine has been shown to increase osteoclastogenesis via p38 pathway-mediated DC-STAMP expression (without affecting NFATc1 signal) [16], and vitamin E (α-tocopherol) reportedly increases osteoclast fusion through p38-mediated transcriptional upregulation of DC-STAMP expression [17]. Therefore, we next evaluated the effect of letrozole on MAPK activation, including ERK, JNK, and p38. The results demonstrated that the phosphorylation status of p-ERK and p-JNK was not affected by letrozole, but that of p38 was markedly suppressed by 10 µM letrozole (Figure 5A). As mentioned above, DC-STAMP is reportedly induced by p38 activation. We postulate that the letrozole-mediated downregulation of DC-STAMP is induced by the reduced activation of p38 in the presence of letrozole

### 2.7. Anti-Osteoclastogenic Effect of Letrozole Was Abolished by p38 Activation

**Figure 6:**
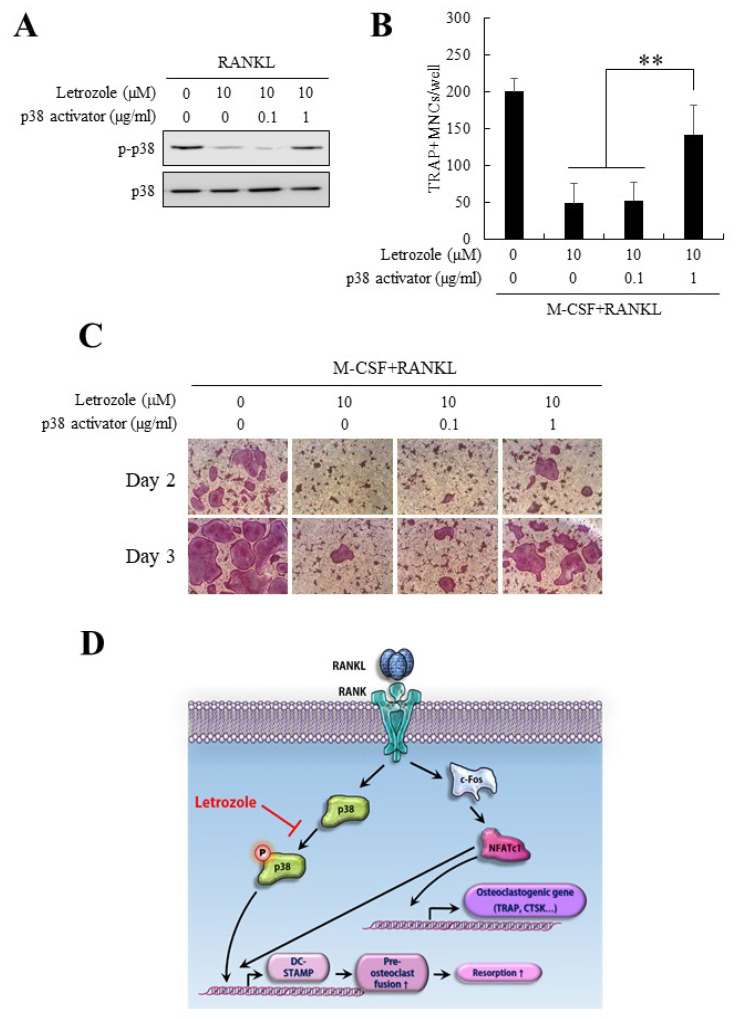
(**A**) Serum-starved BMMs were pretreated with letrozole (10 μM) and the indicated dose of the p38 activator (anisomycin) for 1 h and stimulated with RANKL (100 ng/mL) for 5 min. Whole-cell lysates were evaluated for p-p38 and p38 by western blotting. (**B**) BMMs were differentiated with the osteoclastogenic medium (M-CSF + RANKL) in the presence of indicated combinations of letrozole or p38 activator. On day 3, TRAP-positive multinucleated osteoclasts were enumerated. (**C**) The representative TRAP stained images of (**B**) are shown. (**D**) A schematic diagram for the regulatory mechanism of letrozole in osteoclastogenesis. Letrozole reduces the transcriptional activation of DC-STAMP through the downregulation of p38 activity, which leads to a decrease in pre-osteoclast fusion and subsequent bone resorption. All quantitative data are presented as mean ± standard deviation (SD), ** *p* < 0.01.

As p38 phosphorylation was substantially reduced after letrozole treatment without significant changes in other MAPK pathways, we next induced p38 activation using anisomycin, a well-known p38 activator [18,19], in the presence of letrozole. As shown in Figure 6A, the lowered level of p38 phosphorylation in the presence of letrozole was successfully recovered following treatment with the p38 activator (1 μg/mL). Moreover, the decreased osteoclast differentiation (pre-osteoclast fusion) by letrozole was reversed after treatment with the p38 activator (Figure 6B,C). These results suggested that the anti-osteoclastogenic effect of letrozole was mediated via the downregulation of the p38 pathway (Figure 6D).

## 3. Discussion

To develop into mature osteoclasts with multinucleated morphology, immature mono-nucleated osteoclasts need to meet at least one of these two prerequisites: an increased chance of mutual cell-to-cell contact by accelerated cell mobility or an increased chance of fusion by accelerated cell fusion mechanisms. In this study, letrozole did not influence cell mobility. However, letrozole reduced the average size of mature osteoclasts by reducing osteoclast fusion and resulted in decreased bone resorptive activity (Figure 1C and Figure 2). Among the osteoclast differentiation markers, DC-STAMP showed the declining tendency induced by letrozole. These results suggest that letrozole suppresses cell fusion by downregulating DC-STAMP expression.

DC-STAMP is a 470-amino acid transmembrane protein that plays an essential role in cell fusion between two lipid bilayers and is upregulated through the RANKL-NFATc1 pathway [8,15]. DC-STAMP plays critical roles in the multinucleation of osteoclasts; therefore, DC-STAMP-/- mice show an osteopetrotic phenotype owing to the absence of functional multinucleated osteoclasts [14,20]. DC-STAMP is currently considered another master regulator of osteoclastogenesis in addition to NFATc1 because of its diverse roles in osteoclast fusion, RANKL-ITAM-ITIM signaling, and communication between osteoclasts and osteoblasts [9]. A recent study has revealed that the immunoreceptor tyrosine-based inhibition motif (ITIM) and surrounding amino acids in tails of DC-STAMP play a role in NFATc1 translocation from the cytoplasm to the nucleus [15]. NFATc1 translocation and intracellular Ca^2+^ oscillations are two main prerequisites for activating genes essential for osteoclastogenesis. Although the specific ligand for DC-STAMP is still unknown, DC-STAMP also affects osteoclast differentiation through physical interactions with various proteins, such as connective tissue growth factor (CTGF, CCN2) and peptidyl-prolyl cis-isomerase NIMA-interacting 1 (Pin1) [9,21,22]. CTGF promotes osteoclastogenesis through direct binding to DC-STAMP [21] and Pin1 regulates the expression and localization of DC-STAMP at the plasma membrane level via its isomerase activity [22]. Therefore, we believe that the decreased pre-osteoclast fusion, as well as the osteoclast differentiation in the presence of letrozole, was due to the letrozole-induced downregulation of DC-STAMP expression.

In the present study, the expression level of c-Fos-NFATc1, one of the major pathways for osteoclast differentiation, was not significantly decreased by letrozole. Other pathways, including NF-κB and MAPKs, such as JNK and ERK, were not significantly reduced, with only p38 significantly affected by letrozole, which resulted in the downregulation of DC-STAMP expression. This result is consistent with that of previous studies, showing that the NFATc1-independent alternative expression of DC-STAMP occurs via the p38 pathway [8,16,17]. Importantly, ectopic activation of the p38 pathway ameliorated the anti-osteoclastogenic effect of letrozole (Figure 6).

Although letrozole suppressed the fusion process of osteoclasts, it remained unclear even when it also suppressed the direct osteoclastogenic pathway. Interestingly, letrozole did not affect the expression of NFATc1 and c-Fos but reduced the expression levels of CTR, TRAP, and CTSK, the osteoclast differentiation markers. This ambiguous result can be explained by the following three hypotheses: letrozole did not affect the expression of NFATc1 but may have affected its activity by influencing (i) Ca^2+^ oscillation through the Ca^2+^-dependent calcineurin pathway; (ii) the translocation of NFATc1 regulated by DC-STAMP; (iii) the combinatory activation of microphthalmia-associated transcription factor (Mitf), and PU.1, irrespective of NFATc1 activity. Phosphorylation of p38 evokes Mitf expression, and the interaction between Mitf and NFATc1 is a prerequisite for the expression of TRAP, CTSK, and CTR [23,24,25]. Consequently, suppression of p38 by letrozole can downregulate the expression of TRAP, CTSK, CTR, and DC-STAMP [17,20]. Therefore, it is reasonable to assume that letrozole influences both stages of osteoclastogenesis, which includes the direct osteoclast differentiation stage and the fusion stage of pre-osteoclast cells. These phenomena and hypotheses need to be verified in further studies.

Although our study involved cell-based experiments, letrozole showed anti-osteoclastic activity in vitro. In contrast, the most common side effect of letrozole administration is osteoporosis, and hence, patients are currently recommended bisphosphonates along with letrozole. Indeed, letrozole is known to cause acute osteoporosis; the rate of bone loss is estimated at 2.6% per year when compared with 2% per year observed in normal postmenopausal women [25,26]. We postulate that this contradictory result could be attributed to disregarding osteoblast-related factors in vivo. As osteoclasts communicate with osteoblasts for bone homeostasis via various mechanisms [27,28,29], the suppression of mature osteoclast formation could alter the differentiation or activation of osteoblasts. Therefore, in future studies, the effect of letrozole on osteoblast differentiation in vitro will be evaluated first and then consideration should be given to the effect of letrozole on the mutual regulation between osteoclasts and osteoblasts.

In conclusion, we revealed that letrozole exerts anti-osteoclastogenic effects through the downregulation of the p38/DC-STAMP pathway, which results in reduced bone resorption activity in vitro. Our data will provide another line of evidence that addresses the effects of letrozole on bone homeostasis.

## 4. Materials and Methods

### 4.1. Reagents

Letrozole (4,4-(1H-1,2,4-Triazol-1-ylmethylene) bisbenzonitrile) was purchased from Sigma-Aldrich (St. Louis, MO, USA) and dissolved in dimethyl sulfoxide (DMSO). Recombinant RANKL and M-CSF were obtained from PeproTech (Rocky Hill, NJ, USA), and the antibodies against NFATc1 and c-Fos were purchased from Santa Cruz Biotechnology, Inc. (Santa Cruz, CA, USA). The other antibodies (anti-phosphor-IKK, anti-IKK, anti-phosphor-ERK, anti-ERK, anti-phospho-JNK, anti-JNK, anti-phosphor-p38, and anti-p38) were purchased from Cell Signaling Technology (Danvers, MA, USA). The leukocyte acid phosphatase kit (TRAP staining kit) and antibody for β-actin were obtained from Sigma-Aldrich. All other reagents were purchased from Sigma-Aldrich.

### 4.2. In Vitro Osteoclastogenesis

BMMs were purified and used as precursor cells for osteoclasts, as previously described [30,31]. Briefly, 5-week old ICR mice were sacrificed, and the femora and tibiae were dissected. The animal experiments were performed with approval from the Committee on the Care and Use of Animals in Research, Pusan National University (PNU-2018–2037). Whole bone marrow cells were flushed out with 1 mL of α-modified Eagle Medium (α-MEM; WelGENE Inc., Daegu, Korea) and cultured on 100-mm culture dishes for 1 day. The next day, attached stromal cells were discarded and floating monocytes were harvested and re-plated on Petri dishes in the presence of M-CSF (30 ng/mL) for 3 days. On day 3, the adherent macrophages were detached by mechanical scraping and stored in a deep freezer for further use. BMMs (4 × 10^4^ cells/48-well plates; 2 × 10^5^ cells/6-well plates) were allowed to differentiate into osteoclasts with osteoclastogenic medium (30 ng/mL M-CSF plus 100 ng/mL RANKL) for 4 days. At the end of the culture period, osteoclastogenic differentiation was assessed by TRAP staining, and TRAP-positive giant cells with ≥3 nuclei were regarded as osteoclasts.

### 4.3. MTT Assay

The effects of letrozole on cell viability and proliferation were determined by a standard colorimetric assay using 3-(4,5-dimethylthiazol)-2,5-diphenyltetrazolium bromide (MTT; Sigma-Aldrich). To summarize, BMMs were plated in 96-well plates in the presence of predetermined letrozole concentrations for up to 3 days. On the indicated day (Figure 1A,B), BMMs were further cultured with MTT solution (0.5 mg/mL MTT, dissolved in α-MEM) for 4 h. The intensity of the blue formazan product formed was evaluated using a microplate reader (at 570 nm).

### 4.4. Resorption Pit Formation Assay

To assess the bone-resorbing activities of osteoclasts, BMMs were cultured on commercial dentin discs (Immuno Diagnostic Systems, Boldon, UK) for 8 days in the presence or absence of letrozole (0 μM, 0.1 μM, or 1 μM). At the end of the culture period, adherent osteoclasts were removed by intense washing with 0.5% Triton X-100, and resorption pits were stained using a 1% toluidine blue solution. The dentin discs were observed under a light microscope and the resorbed area was calculated using Zeiss LSM Image Browser software (ver 3.0, Oberkochen, Germany).

### 4.5. Trans-Well Migration Assay

In BMMs, cell migration was determined using the Boyden chamber system. Briefly, BMMs were seeded in the upper compartment of the trans-well chamber (0.8 μm pore size; Costar, Pleasanton, CA, USA) in M-CSF (30 ng/mL), containing serum-free media, and allowed to migrate to the lower side. After 16 h, the filter membrane was fixed and stained with hematoxylin. The BMMs that migrated to the lower side of the membrane were photographed using a light microscope, and the numbers of BMMs were quantified.

### 4.6. RT-PCR and Quantitative Real-Time PCR Analyses

Total RNA was purified using TRIzol Reagent (Invitrogen, Waltham, MA, USA), and 1.5 μg of RNA was reverse-transcribed under standard conditions with Superscript II (Invitrogen). The PCR reaction was performed with 1 μg of synthesized cDNA using the following primer sets: DC-STAMP, 5′-TGGAAGTTCACTTGAAACTACGTG-3′ (forward) and 5′-CTCGGTTTCCCGTCAGCCTCTCTC-3′ (reverse); TRAP, 5′-ACTTCCCCAGCCCTTACTACCG-3′ (forward) and 5′-TCAGCACATAGCCCA-CACCG-3′ (reverse); CTR, 5′-TGCATTCCCGGGATACACAG-3′ (forward) and 5′-AGGAACGCAGACTTCACTGG-3′ (reverse); CTK, 5′-AGGCGGCTATATGACCACTG-3′ (forward) and 5′-CCGAGCCAAGAGAGCATATC-3′ (reverse); actin, 5′-CGATGCCCTGAGGCTCTTTT-3′ (forward) and 5′-GGGCCGGACTCATCGTACTC-3′ (reverse). For quantitative real-time PCR analysis, 2 μg of cDNA was mixed with SYBR green PCR master mix (Applied Biosystems, Forster, CA, USA) and amplified for 40 cycles in AB7500 instruments (Applied Biosystems). The DC-STAMP gene expression level was determined by that of actin using the 2^−∆∆Ct^ method. The primer sequences used in real-time PCR were as follows: DC-DTAMP, 5′-GGGTGCTGTTTGCCGCTG-3′ (forward) and 5′-CGACTCCTTGGGTTCCTTGCT-3′ (reverse); actin, 5′-CGATGCCCTGAGGCTCTTTT-3′ (forward) and 5′-GGGCCGGACTCATCGTACTC-3′ (reverse).

### 4.7. Western Blotting

Western blotting was performed following a standard protocol. In brief, cultured BMMs were lysed in lysis buffer (50 mM Tris; pH 8.0, 150 mM NaCl, 0.5% sodium deoxycholate, 5 mM EDTA, 1% Triton X-100, and complete protease inhibitor cocktail), and protein lysates (30 μg) were subjected to SDS-PAGE. In the case of multiple blots using phosphor-specific antibodies, equal amounts of divided lysates were individually subjected to SDS-PAGE. The electrophoretically separated proteins were transferred onto nitrocellulose membranes and blocked with 5% skim milk for 1 h. The membranes were incubated with appropriate primary antibodies (4 °C overnight) and secondary antibodies (for 1 h at room temperature), followed by development with chemical luminescence reagents. Actin and non-phosphor proteins served as loading controls.

### 4.8. Statistics

Unless otherwise specified, all data were obtained from at least three independent experiments conducted in triplicate. To determine the statistical significance between any two groups, the Student’s *t*-test was used. Differences with *p* < 0.01 were considered significant and are shown with asterisks (** *p* < 0.01; *** *p* < 0.001).
